# History of Heart Transplantation: a Hard and Glorious Journey

**DOI:** 10.21470/1678-9741-2017-0508

**Published:** 2017

**Authors:** Noedir A. G. Stolf

**Affiliations:** 1 Heart Institute of Hospital das Clínicas of Faculdade de Medicina of Universidade de São Paulo (InCor-HCFMUSP), São Paulo, SP, Brazil.

## INTRODUCTION

This year we celebrate 50 years of the first interhuman heart transplantation. So, I believe that it is interesting to review the most important steps of this glorious journey.

Heart transplantation first performed in the course of experiments of other nature in the beginning of 20^th^ century, seen as a speculation for the future in the middle of the same century, is now widely accepted by medical and lay communities as a valuable therapeutic procedure.

This history can be divided into an experimental and a clinical heart transplantation periods, with the first one in heterotopic non-auxiliary, heterotopic auxiliary and orthotopic transplantations.

## Heterotopic Non-Auxiliary Heart Transplantation

The first heart transplant was performed by Alexis Carrel and Charles Guthrie in 1905 at the University of Chicago^[1]^. In the course of experiments to develop the technique of vascular anastomoses, they have done limb reimplantation, thyroid gland as well kidney and heart transplantation. The description of their heterotopic heart transplantation is as follows: "The heart of a small dog was extirpated and transplanted into the neck of a larger one by anastomosing the cut ends of the jugular vein and the carotid artery to the aorta, the pulmonary artery, one of the venae cavae and a pulmonary vein. The circulation was reestablished through the heart, about an hour and 15 minutes after the cessation of the beat; 20 minutes after the reestablishment of the circulation, the blood was actively circulating through the coronary system. A small opening being made through the wall of a small branch of the coronary vein, an abundant dark hemorrhage was produced. Then strong fibrillar contractions were seen. Afterward contractions appeared and about an hour after the operation, effective contractions of the ventricles began. The transplanted heart beat at the rate of 88 per minute, while the rate of the normal heart was 100 per minute. A little later tracing was taken. Owing to the fact that the operation was made without aseptic technique, coagulation occurred in the cavities of the heart after about two hours, and the experiment was interrupted".

**Fig. 1:**
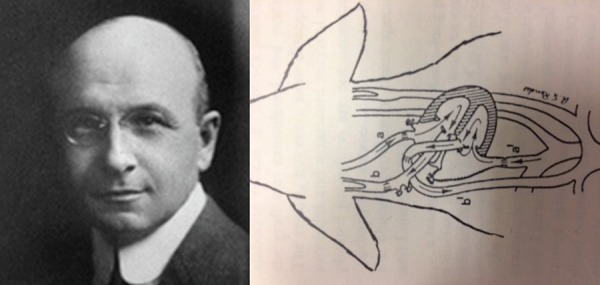
On the left, the elegant Alexis Carrel. On the right, the probable anastomotic arrangement: a= distal carotid artery; a'= proximal carotid; b= distal jugular vein and b'= proximal jugular vein.

Although the exact details of experiments were not known the most probable arrangement of anastomosis are shown in Figure 1 adaptation in a book by Najarian and Simmons^[2]^. The problem with this model is that arterial inflow is in the atrium and aortic outflow is under venous pressure, in other words, totally not physiological. Despite these problems, the experiment demonstrated that heart transplantation was possible, that the heart separated of its blood supply could be sutured to the circulation of other animal and recover normal function.

The next reference to transplantation of heart in dogs was made by Mann et al.^[3]^ in 1933. The objective of the experiments was to obtain a totally denervated heart. In one of their models, the heart transplanted in the neck had pulmonary artery anastomosed to proximal jugular vein and aorta to distal carotid artery with a more adequate coronary perfusion. They obtained a longest survival of eight days and mean survival of four days. They made observations of recipient donor heart different response of rate and furthermore studied the histology of rejection, and as Carrel stating that surgical technique is not the cause of failure of the grafted heart.

More than two decades later, Marcus et al.^[4]^ working at the Chicago Medical School, using a modified Mann technique, studied complicated methods of cardiac preservation with poor survival. In 1953, Marcus et al.^[5]^ again modified the Mann preparation, so that the donor left ventricle would act as a pump. The proximal end of the divided recipient common carotid artery was anastomosed to the donor left atrium and the recipient distal common carotid to the donor innominate artery, so that the donor left ventricle supplied blood to its own coronary arteries and to the recipient cerebral circulation. Maximum survival with this technique was 48 hours. Longer survival were obtained later on with heterotopic, non-functioning heart transplant.

The heterotopic abdominal and neck technique is a valuable tool for studies of immunosuppressors or other studies on the graft^[6]^. In 1962, Demikhov^[7]^ published an extensive work he developed in Russia. Among his achievements, there are reports of heterotopic heart transplantation in the inguinal region in 1940, heterotopic intrathoracic heart transplants in 1946. Twenty-four variants were described and he mentions a 32 days survival of the graft.

**Fig. 2:**
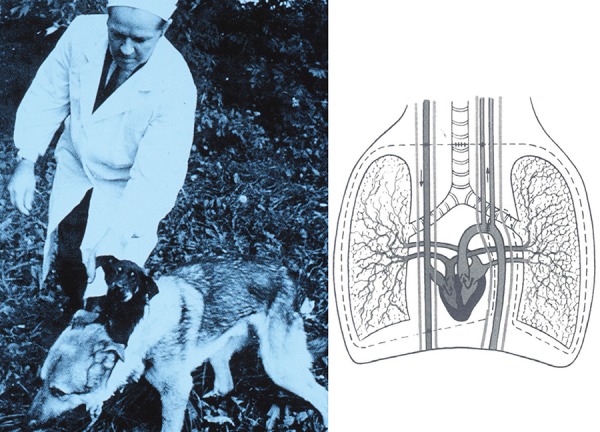
On the left, Vladimir Demikhov and a dog with two heads. On the right, an intrathoracic heart-lung transplant.

Another technical amazing achievement by Demikhov was the transplantation of an additional head in a dog and the intrathoracic heart - lung transplantation before the availability of cardiopulmonary bypass (Figure 2). Differently than previous authors, Demikhov considered that the failure of grafts was due to surgical technical factors.

## Heterotopic Auxiliary Heart Transplantation

In this field, Demikhov^[7]^ performed 22 canine intrathoracic auxiliary heart transplants between 1951 and 1955. After grafting, he excluded the recipient heart from circulation by ligating the great vessels and mitral annulus. One such animal awoke from anesthesia stood up, drank but died 15 hours later.

This experiment was the first one to demonstrate that transplanted heart could assume total circulation and the death of the dog was attributed to superior vena cava thrombosis.

In 1989, at the International Society for Heart-Lung Transplantation Annual Meeting, in Munich, the Organizing Committee could find Dr. Demikhov in the country of Russia and payed a tribute to him at Gala Dinner. Apparently, he was bright and could not understand or speak a word in English, nevertheless, we could shake his hands.

Reemtsma group, in 1964 and later in 1966^[8]^, studied also intrathoracic auxiliary transplanted canine hearts with immunosuppression with a 4-hour survival with the recipient heart fibrillated. Another group, Johansson, Soderlund and William-Olsson^[9]^ working at the Karolinska Institute in Sweden performed intrathoracic auxiliary heart transplantation for assistance of the recipient left heart maintaining cardiac output for one hour.

## Orthotopic Heart Transplantation

By the beginning of 1950, there was evidence of the feasibility of heart transplantation and recovery of adequate function of grafted heart. The step forward was the experimental orthotopic heart transplantation. In this phase, the major concern was the maintenance of the recipient animal, the preservation of the graft and the development of a surgical technique to minimize ischemia. In 1953, Neptune et al.^[10]^ faced all these problems in their experiments. Their option was to use topical hypothermia for both donor and recipient and to avoid multiple venous anastomoses they performed heart-lung transplants. Three dogs survived six hours.

In 1957, Webb and Howard^[11]^ reported preservation of the heart in dogs by flushing the organ with cold potassium citrate and successfully transplant heterotopically. So, these authors in a certain way, opened the pathway for long distance procurement.

In 1959, Goldberg et al.^[12]^ at the University of Maryland performed the first orthotopic heart transplantation in dog. They created the technique of left atrial anastomosis with a cuff of the orifices of the four pulmonary veins the conventional technique of cardiac transplantation that was used routinely for decades. The anastomosis of great vessels was performed by sutures and both vena cava were connected with methacrylate tubes. Recipient was maintained with cardiopulmonary bypass, but surprisingly the donor heart was arrested with potassium citrate in normothermia. The longest survival was 20 minutes.

**Fig. 3:**
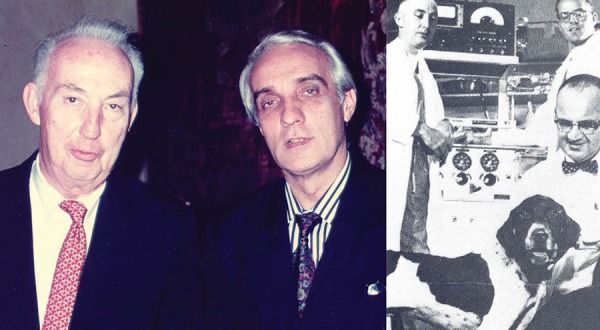
On left, Dr. Shumway and I. On the right, Stanford University experimental laboratory. Standing, Dr. Schumway and Richard Lower, Dr. Stofer, a veterinarian that was also chief of clinical cardiopulmonary bypass with a transplanted dog.

Webb et al. in 1959^[13]^ performed orthotopic heart transplantation in dogs with hypothermic preservation but individual pulmonary veins connection. Ten dogs survived from 30 to 450 minutes. Subsequently, in the same year, Cass and Brock^[14]^ working at the Guy's Hospital in London, performed autotransplantation and homologous transplantation using the left atrial cuff technique described by Goldberg and introducing the right atrial anastomosis instead of caval anastomosis. In other words, they established the standard technique of orthotopic heart transplantation used clinically until introduction of bicaval technique. In this scenario, late 1950, Dr. Shumway group, at Stanford, started to perform orthotopic heart transplantation in dogs with cardiopulmonary bypass, preservation of the donor heart by immersion for 5 minutes in a in 4°C saline. They standardized surgical technique incorporating the previous concepts of left and right atrial cuffs. Lower and Shumway^[15]^ published their experience in 1960 reporting that five of eight animals survived for 6-21 days without immunosuppression. In sequence, Lower et al.^[16]^ made an important contribution in 1965, showing a decrease of electrocardiography voltage in rejection and the reversal of process with a combination of azathioprine and methylprednisolone achieving a survival of 250 days (Figure 3).

## Clinical Heart Transplantation

**Fig. 4:**
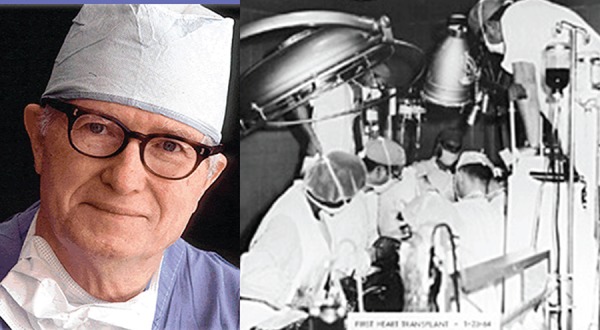
On the left, Dr. James Hardy and, on the right, a photograph of the operating room of the first human xenotransplantation.

**Fig. 5:**
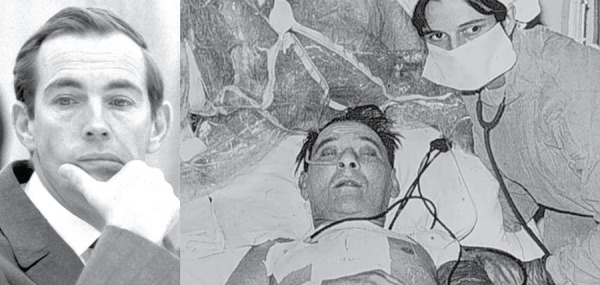
On the left, Dr. Barnard and, on the right, Louis Washkansky, the first recipient.

**Fig. 6:**
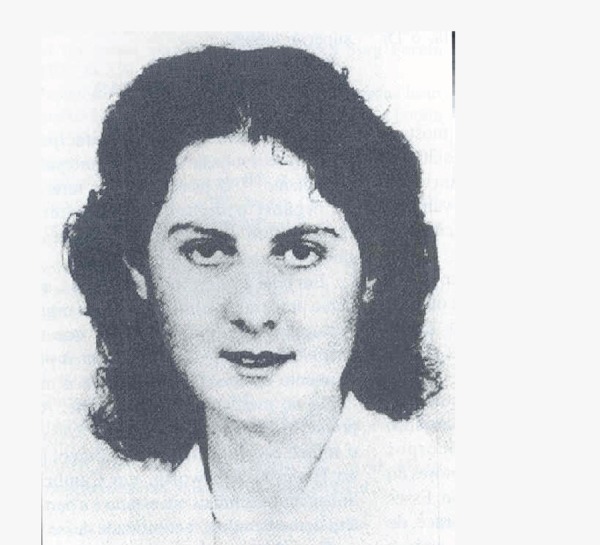
Denise Darvall, the first donor for a heart transplant

By the mid - 1960 solutions for the key problems were reasonably addressed in the experimental laboratory. Recipient support, myocardial protection, function of the transplanted organ, diagnosis and treatment of rejection seem to be solved remaining the ethical and legal aspects. In 1964, Hardy et al.^[17]^ working at the University hospital in Jackson, Mississippi felt justified to move to human heart transplantation. As the concept of brain death was not stablished, this group discussed all the legal ethical issues considering that they have to wait for cardiorespiratory arrest and not interrupting mechanical ventilation of the donor, thus, the possibility that the donor would have cardiac arrest and recipient would be in serious decompensation would be slim. A second potential recipient was admitted, actually, he was an extremely bad case, in cardiogenic shock, leg amputation, tracheostomy and finally put on cardiopulmonary bypass. It was decided to use a large chimpanzee as the donor. On January, 23^rd^, 1964 Dr. Hardy^[17]^ performed the first human transplantation, actually, a xenotransplant. The transplant was unable to maintain the circulatory load and the patient died one hour later (Figure 4). On December, 3^rd^, 1967, the medical and lay people were amazed by the news of first interhuman heart transplantation. At the Groote Schuur Hospital, in Cape Town, South Africa. Dr. Christiaan Nethling Barnard performed this heart transplant^[18]^. The recipient Louis Washkansky, a 54-year-old man in end stage ischemic cardiomyopathy received the heart of a girl, Denise Darvall hit by a drunk driver, became the first human heart donor. They stopped ventilation, waited for cardiac arrest and then cardiopulmonary bypass was initiated to resuscitate the heart. After an initial excellent recovery, the patient died on the 18^th^ postoperative day (Figures 5 and 6). Three days later, Adrian Kantrowitz performed the 2^nd^ transplant, a pediatric one^[19]^. The donor was an anencephalic baby and the recipient was 18-day-old child with Ebstein anomaly. The operation was performed at the Maimonides Hospital, Brooklyn, New York and the patient survived six hours^[19]^. The fourth heart transplant was performed at Stanford, January, 6^th^, 1968 in an adult patient.

**Fig. 7:**
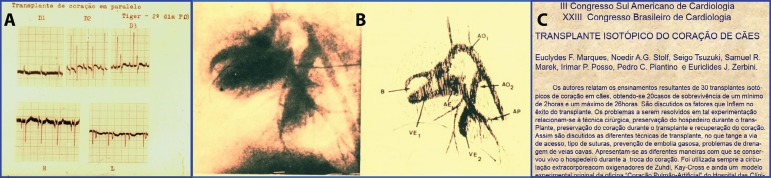
In A, an electrocardiography and, in B, angiography of an intrathoracic heterotopic heart transplant. In C, an abstract of a paper presented by our team.

**Fig. 8:**
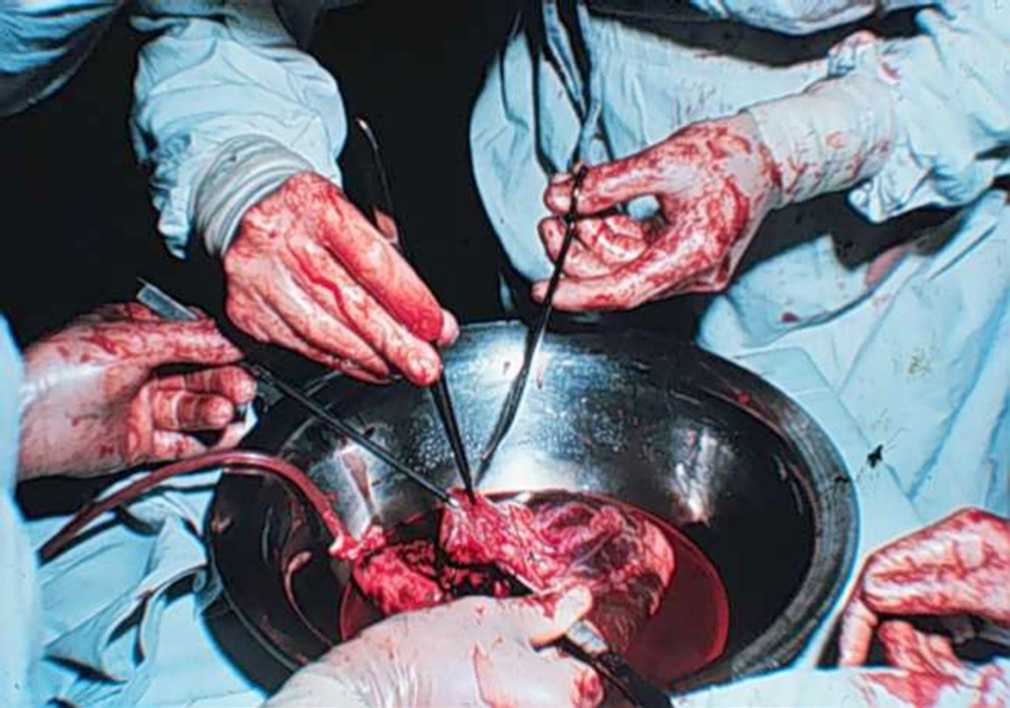
Photograph of preparation of the donor for the first Brazilian heart transplant with continuous normothermic blood perfusion.

**Fig. 9:**
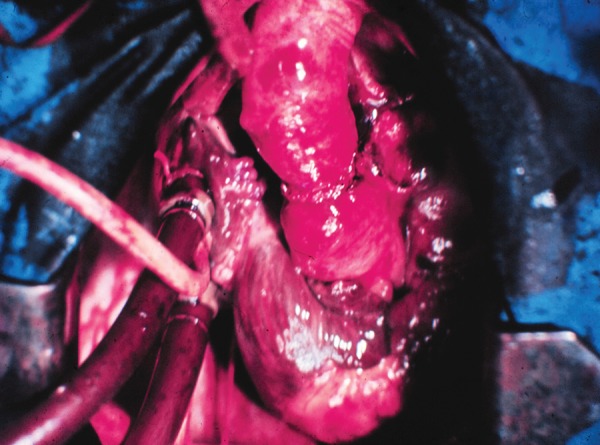
Final aspect of first Brazilian heart transplant.

The great interest of cardiovascular surgeons for this challenging surgical procedure is testified by the fact that at the end of 1968, 102 transplants were made in 17 countries and 52 centers. In Brazil, heart transplant has been performed in dogs by Marques and others. The enthusiasm of this group led to Marques and Stolf (then a medical student) to propose to Prof. Zerbini the performance of human transplant in 1964. It was considered a premature indication (Figure 7). After Barnard's successful procedure, Prof. Zerbini decided to prepare the group to perform clinical heart transplantation. Preparation included cardiologists, surgeons and a hematologist serving also as an immunologist. On May, 26^th^, 1968 the transplant took place, the recipient was a patient with dilated cardiomyopathy and the donor, a patient with severe cerebral trauma. The donor was in an adjacent room to the recipient, had electrocardiography and electroencephalography monitoring. The brachiocephalic trunk was cumulated, ventilation turned off. When heart stopped, perfusion of the heart was started through the cannula, the heart was harvest and transferred to the recipient room . Transplantation was performed with continuous normothermic perfusion of the heart (Figure 8 and 9). The patient had an excellent recovery and died on the 28^th^ postoperative day from rejection. The second patient with ischemic cardiomyopathy had a little more than one-year survival in excellent clinical condition. The third patient, also with ischemic cardiomyopathy and diabetes, died on the 60^th^ day from infection starting in the femoral artery cannulation site.

**Fig. 10:**
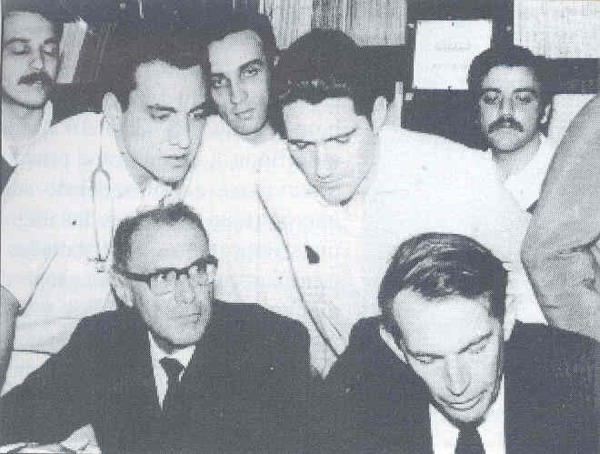
In 1969, photograph of the visit of Christiaan Barnard to University of São Paulo Medical Center. Sitting on the left, Dr. Zerbini and, on the right, Dr. Barnard observing an electrocardiogram tracing of a transplanted patient.

On July, 13-16^th^, 1968 Dr. Barnard organized a meeting called "Experience with human heart transplantation", in Cape Town, South Africa. Sixteen guest speakers were invited; three from South Africa; four from USA; two from England; one from India; one from Argentina; 1 from Chile and Dr. Zerbini. Brazil was included among the pioneer countries (Figure 10).

This meeting resulted in a very interesting publication: "The Proceedings of this Symposium". It is extremely interesting to read this book. All participants commented on the subjects. These comments go from silly to wise observations. Worldwide in 1969, 50 patients were transplanted; in 1970, less than 20 and in 1971, 10 patients.

On April, 4^th^, 1969, Denton Cooley implanted the first total artificial heart. It was a device that was being tested by Dr. Domingo Liotta, from Argentina, in Dr. DeBakey laboratory across the street. The patient was kept in mechanical circulatory assistance for three days and then transplanted. It was the first bridge to transplantation, but the patient died two days later. The 1970 decade is what I call the disenchantment period. Virtually, only four centers kept their heart transplantation programs: the Groote Schuur in Cape Town under the leadership of Marius Barnard (Christian brother); Stanford-Shumway group; Richard Lower-Richmond-Virginia (after leaving Stanford) and Christian Cabrol-La Pitié Salpetrieri in Paris. Nevertheless, in this period, we had several important achievements mostly at Stanford. The introduction of immunological monitoring, important in azathioprine/steroids era and the development of endomyocardial biopsy by Phillip Caves, both at Stanford. The bioptome was developed based on the Sakakibara clamp and made by Schultz. Schultz was a little old nice man. In 1977, when I spent three months and a half at Stanford following the transplantation program, I went to Schultz home and bought a bioptome manufactured in his garage. With this bioptome, I performed almost 400 biopsies in several cardiomyopathies and transplanted patients.

In 1978, cyclosporine was introduced in kidney transplantation and, in 1980, in cardiac transplantation at Stanford. In 1980, the modern era of heart transplantation started with rapid increase of annual number of transplant centers and number of transplanted patients. It is amazing to look back and see such progress in an 80-year period.
